# Robust Magnetized Graphene Oxide Platform for In Situ Peptide Synthesis and FRET-Based Protease Detection

**DOI:** 10.3390/s20185275

**Published:** 2020-09-15

**Authors:** Seongsoo Kim, Sang-Myung Lee, Je Pil Yoon, Namhun Lee, Jinhyo Chung, Woo-Jae Chung, Dong-Sik Shin

**Affiliations:** 1 Division of Chemical and Bioengineering, Kangwon National University, Gangwon-do 24341, Korea; ksswdf@kangwon.ac.kr (S.K.); smlee@cantis.co.kr (S.-M.L.); yjp@boditech.co.kr (J.P.Y.); nh131@kirams.re.kr (N.L.); 2 Department of Research and Development, Cantis Inc., Ansan-si, Gyeonggi-do 15588, Korea; 3 Department of Integrative Biotechnology, Sungkyunkwan University, Suwon 16419, Korea; endeavor10@skku.edu; 4 Department of Chemical and Biological Engineering, Sookmyung Women’s University, Yongsan-gu, Seoul 04310, Korea; 5 Industry Collaboration Center, Sookmyung Women’s University, Yongsan-gu, Seoul 04310, Korea

**Keywords:** magnetic graphene oxide (MGO), proteases, in situ peptide synthesis, fluorescence resonance energy transfer (FRET), biological assays

## Abstract

Graphene oxide (GO)/peptide complexes as a promising disease biomarker analysis platform have been used to detect proteolytic activity by observing the turn-on signal of the quenched fluorescence upon the release of peptide fragments. However, the purification steps are often cumbersome during surface modification of nano-/micro-sized GO. In addition, it is still challenging to incorporate the specific peptides into GO with proper orientation using conventional immobilization methods based on pre-synthesized peptides. Here, we demonstrate a robust magnetic GO (MGO) fluorescence resonance energy transfer (FRET) platform based on in situ sequence-specific peptide synthesis of MGO. The magnetization of GO was achieved by co-precipitation of an iron precursor solution. Magnetic purification/isolation enabled efficient incorporation of amino-polyethylene glycol spacers and subsequent solid-phase peptide synthesis of MGO to ensure the oriented immobilization of the peptide, which was evaluated by mass spectrometry after photocleavage. The FRET peptide MGO responded to proteases such as trypsin, thrombin, and β-secretase in a concentration-dependent manner. Particularly, β-secretase, as an important Alzheimer’s disease marker, was assayed down to 0.125 ng/mL. Overall, the MGO platform is applicable to the detection of other proteases by using various peptide substrates, with a potential to be used in an automated synthesis system operating in a high throughput configuration.

## 1. Introduction

Graphene oxide (GO) is a two-dimensional, atomically thin carbon material with good physisorption for diverse molecules, high water dispersibility, facile surface modification capability, and high biocompatibility [1,2,3,4,5,6,7,8,9,10]. Various chemicals can be immobilized on functionalized GO through the use of amide bonding, diazonium salts, atom transfer radical polymerization (ATRP), or click chemistry [11,12,13,14,15,16]. In addition, graphene-based materials are known as energy acceptors in energy transfer reactions due to their peculiar electronic properties [17]. As photophysical calculations have revealed energy transfer phenomena, GO can be used as a quencher (acceptor) of electronic excited states in fluorescent dyes (donors) resulting in the phenomenon of fluorescence resonance energy transfer (FRET) [18,19,20,21].

The FRET combination of GO and fluorescent dyes or luminescent nanoparticles has been utilized as a promising biochemical analysis platform. The fluorescence ‘turn-on’ phenomenon induced by the structural truncation or deformation of probe molecules on GO after target recognition or binding has been widely applied to the sensitive detection of small molecules, biomolecules (including metal ions (e.g., K^+^, Hg^2+^, and Pb^2+^)), proteins, and DNA [22,23,24,25,26,27,28,29]. Recently, GO/peptide complexes were used to detect proteolytic activity by observing the turn-on phenomena of the quenched fluorescence induced by the release of fluorescently labeled peptide fragments from the GO surface [30,31]. However, the purification and isolation steps are quite cumbersome when GO is applied as a biosensor or an assay tool due to the associated nano- or micro-sized dimensions and the high water dispersibility of GO. Thus, Zhou et al. have reported that magnetized GO-based colorimetric biosensors conveniently manipulate samples for the aptamer-mediated detection of ochratoxin A [32]. The introduction of magnetism to the GO biosensor platform eliminated the need for tedious and high-cost chemical modification processes.

For the development of a GO platform to detect disease biomarkers such as proteases, it is essential to not only design optimal specific sequences of synthetic peptide molecules but to also employ a strategy for their immobilization on the GO platform to ensure high stability and proper orientation. Previous reports generally utilized pre-synthesized peptides, which were then physically/chemically immobilized onto the GO surface [33,34]. This strategy can suffer from limitations, including low stability to interference by non-specific target molecules and possible coupling reactions at random sites of peptides that may lead to a loss of reactivity toward the target molecule.

Here, for the first time, robust FRET peptide MGO (magnetic GO) biosensors are introduced. In situ sequence-specific peptide syntheses were performed with the advantage of simple purification and isolation. This was followed by FRET-based assays for determining the proteolytic activities of biologically meaningful proteases such as trypsin, thrombin, and β-secretase. The carboxylic acid-modified MGO nano-platform was prepared through a reaction with chloroacetic acid under basic conditions; magnetization was achieved by the co-precipitation of an aqueous iron precursor solution. After verification of the feasibility of conventional solid-phase peptide synthesis based on Fmoc/*t*-Bu chemistry in an organic solvent on an MGO matrix, the optimal length of polyethylene glycol (PEG) spacers for the FRET-based protease assays was thoroughly investigated. Upon the synthesis of three different peptides specific to proteases, such as trypsin (KKK), thrombin (LVPRGS), and β-secretase (EVNLDA) on the MGO matrices, FRET-based protease assays were evaluated with the FRET peptide MGO platform.

## 2. Materials and Methods

### 2.1. Materials

Graphite flakes was purchased from Alfa Aesar (Ward Hill, MA, USA). *N*,*N*-dimethylformamide (DMF), *N*-methyl-2-pyrrolidone (NMP), dimethyl sulfoxide (DMSO), fluorescein 5-isothiocyanate (FITC), trypsin, thrombin, triisopropylsilane (TIS), *N*,*N*-diisopropylethylamine (DIPEA), trifluoroacetic acid (TFA), *O*-(2-aminopropyl)-*O*′-(2-methoxyethyl)polypropylene glycol 300 (PEG_300_ diamine), *O*-(2-Aminopropyl)-*O*′-(2-methoxyethyl)polypropylene glycol 500 (PEG_500_ diamine), *O*-(2-Aminopropyl)-*O*′-(2-methoxyethyl)polypropylene glycol 800 (PEG_800_ diamine), 1-ethyl-3-[3-dimethylaminopropyl]carbodiimide (EDC), and *N*-hydroxy succinimide (NHS) were purchased from Sigma–Aldrich (St. Louis, MO, USA). Fmoc-photolabile linker (Fmoc-PCL: 4-{4-[1-(9-fluorenylmethyloxycarbonyl-amino)ethyl]-2-methoxy-5-nitrophenoxy}butanoic acid) was purchased from Advanced ChemTech (Louisville, KY, USA). All Fmoc-protected amino acids were purchased from BeadTech (Seoul, Korea).

Fluorescence emission spectra were obtained with a fluorescence spectrophotometer (FluoroMate FS-2, SCINCO, Seoul, Korea). UV/Vis absorption spectra were recorded with a UV/Vis spectrophotometer (OPTIZEN α, MECASYS, Daejeon, Korea). Hysteresis loops were obtained with a vibrating sample magnetometer (Lake Shore #7300, Lake Shore Cryotronics, Westerville, OH, USA). Optical images were acquired with a fluorescent microscope (Zeiss Axio Observer. A1, ZEISS, Oberkochen, Germany). FT-IR spectra were obtained by Fourier transform infrared spectroscopy (FTLA2000-104, ABB, Zurich, Switzerland). Mass spectra of peptides were obtained by MALDI-TOF MS) (Voyager DE STR, Applied Biosystems, Waltham, MA, USA). A UV illuminator for peptide release was purchased from Uvitec (LF-206, 365 nm, 15W, Cambridge, UK).

### 2.2. Synthesis of Graphene Oxide (MGO)

Graphene oxide was obtained from graphite via the improved Hummer’s method [35]. Briefly, NaOH (1.2 g) was added to an aqueous solution (10 mL) of graphene oxide (10 mg) and dispersed under sonication for 2 h. Chloroacetic acid (1 g) was then added to the mixture, followed by sonication for 2 h. The resulting solution (2 mL) was added to a mixture of FeCl_2_·4H_2_O (11 mg) and FeCl_3_·6H_2_O (30 mg) in DI water (18 mL). After stirring the mixture for 2 h at RT, NH_4_OH (1 mL) was added dropwise to the solution and the mixture was stirred for another 1 h. The final product, denoted as MGO and obtained by magnetic separation, was sequentially washed with distilled water and ethanol. The obtained MGO was characterized by Fourier transform infrared spectroscopy (FT-IR), UV/Vis spectra, optical microscopy, and vibrating sample magnetometer (VSM) data.

### 2.3. In Situ Peptide Synthesis on MGO Matrix

Prior to the peptide synthesis, PEG diamines were coupled on the MGO matrix. After dispersing MGO (3 mg) in 5 mL of EtOH, 60 mM of PEG_x_ diamine (x = 300, 500, 800, respectively), EDC (52 mg), and NHS (138.1 mg) were sequentially added, and the mixture was stirred for 6 h. After the reaction, this solution was washed three times with EtOH and NMP under magnetic separation. Swollen PEG–MGO (3 mg) complex was placed into a solution of Fmoc-photolabile linker (1.5 equiv), HBTU (1.5 equiv), and DIPEA (4.0 equiv) in DMF (3 mL) and incubated for 2 h at RT. To acetylate the remaining amine groups of the resin, the resin was treated with a solution of acetic anhydride (150 µL) and DIPEA (150 µL) in DMF (2.7 mL) for 2 h at RT. The peptides were synthesized on the PEG–MGO complex by repeating the coupling and deprotection steps at RT: (a) the coupling step; the resin was treated with a solution of Fmoc-amino acids (3.0 equiv) (e.g., for the sequence of EVNLDA: Fmoc-Ala-OH, Fmoc-Asp(O*t*Bu)-OH, Fmoc-Leu-OH, Fmoc-Asn(Trt)-OH, Fmoc-Val-OH, and Fmoc-Glu(O*t*Bu)-OH, sequentially), HBTU (3.0 equiv), HOBt (3.0 equiv), and DIPEA (6.0 equiv) in DMF (10 mL) for 2 h. (b) the deprotection step; the PEG-MGO complex was treated twice with 20% (*v*/*v*) piperidine/DMF solution (3 mL) for 5 and 10 min, respectively. The PEG–MGO was confirmed by the Kaiser ninhydrin test for the completion of synthetic amino acid for each coupling step. The PEG–MGO complex was washed with DMF, DCM, and MeOH three times after every coupling and deprotection step. For the preparation of the FRET MGO, the peptide-coupled MGO (9 mg) was incubated in a solution of FITC (0.12 mmol) and DIPEA (0.24 mmol) in DMSO (3 mL) for 1 h at RT. The side chain protecting groups of amino acids were fully deprotected by a solution of TFA/TIS/water (94:5:1, 3 mL) for 30 min at RT. For the characterization of peptide synthesis, the peptide on the MGO was released by the UV illuminator for 10 min and the mass spectra of released peptides were obtained by MALDI-TOF.

### 2.4. Evaluation of Proteolytic Activities Using the MGO FRET Platform

For optimization of the PEG length, MGO-diamine PEG_x_-KKK-FITC (x = 300, 500, 800) was incubated with various concentrations of trypsin (0, 5, 10, 20, 40, 80, and 160 μg/mL) in Tris–HCl buffer (20 mM, pH 7.4) for 30 min. After the reaction was completed, the MGO was removed by magnetic separation. Fluorescence of the supernatant was recorded at an excitation of 480 nm and an emission of 514.5 nm. Protease activities were evaluated with corresponding peptides sequences (FITC-LVPRGS-PEG_800_-MGO for thrombin and FITC-EVNLDA-PEG_800_-MGO for β-secretase). The peptide-immobilized MGO was incubated with a serially diluted protease solution starting from 8 µg/mL in Tris-HCl buffer (20 mM, pH 7.4) at 37 °C for 30 min. For the kinetic measurement, the MGO sample was incubated with 2 µg/mL of protease solution for up to 60 min.

## 3. Results and Discussion

### 3.1. Preparation and Characterization of Magnetic GO (MGO)

**Figure 1:**
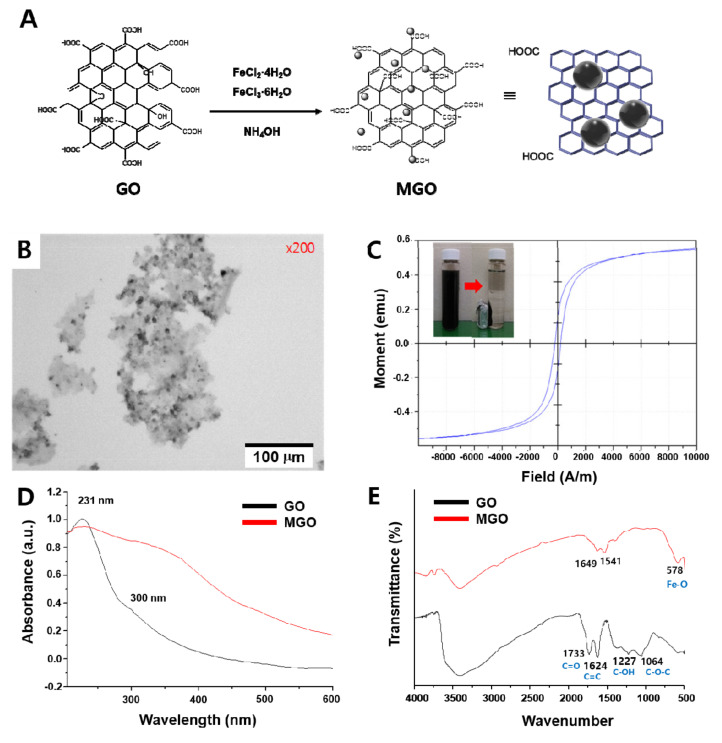
Preparation and characterization of graphene oxide (GO) and magnetic graphene oxide (MGO). (**A**) Schematic of MGO synthesis procedure. (**B**) Optical microscopy image of MGO. (**C**) Magnetic hysteresis loop of MGO. (**D**) UV/Vis absorption spectra of GO and MGO. (**E**) FT-IR spectra of GO and MGO.

Graphene oxide (GO) was prepared from graphite according to the improved Hummers’ method [35]. The GO was then modified with chloroacetic acid to introduce a large amount of carboxyl groups on it [36]. Carboxylic acid groups facilitate the formation of magnetic Fe_3_O_4_ particles on the GO by attracting Fe^2+^ and Fe^3+^ ions close to GO surfaces as well as consolidating the adhesion of Fe_3_O_4_ particles; they also serve as reactive sites for subsequent couplings with the PEG spacer, photocleavable linker, and amino acids to build up diverse protease-specific peptides on the MGO platform. Magnetic Fe_3_O_4_ particles were successfully synthesized on the carboxylated GO surface by the co-precipitation of Fe^2+^ and Fe^3+^ in aqueous solution (Figure 1A) [37]. The MGO was characterized by an optical microscopy, a VSM data, and UV/Vis and FT-IR spectroscopies. From the microscopy image in Figure 1B, the irregular shape of the GO (~200 μm) and dark magnetic microparticles (1–10 μm) bound on the GO surface (gray) are clearly visible. Figure 1C displays the magnetic properties of the prepared MGO. Typical superparamagnetic behavior was revealed by the magnetization loop of the MGO, which showed little hysteresis, remanence (0.144 emu/mg), and coercivity. The inset photograph in Figure 1C shows that the MGO can be easily separated from the aqueous solution in under 1 min with an external magnet and easily re-dispersed in the solution by simple shaking after removing the magnet. Two characteristic peaks appear in the GO UV/Vis spectrum of Figure 1D. Typically, a peak with a maximum at ~231 nm corresponds to π→π* transitions of aromatic C-C bonds, while a broad peak at 320–380 nm can be assigned to Fe-O bonds [38]. After magnetization, the maximum peak (~231 nm) of the GO exhibited a slight red shift to ~245 nm and the absorbance band of the MGO became broader than that of the GO over the entire UV/Vis wavelength range. The FT-IR spectra of both the GO and MGO were also compared. As shown in Figure 1E, the peak at 1733 cm^−1^ corresponding to C=O groups of GO carbonyl and carboxyl moieties shifted to 1649 cm^−1^ due to the formation of carboxylate after the introduction of Fe_3_O_4_ particles on the GO. Similarly, the peak at 1624 cm^−1^ corresponding to skeletal vibrations of the GO domains (C=C) also shifted to 1541 cm^−1^ [39]. The C–O stretching peak corresponding to alkoxy groups (1227 cm^−1^) and the C–O–C stretching peak corresponding to epoxy groups (1064 cm^−1^) disappeared. Instead, the characteristic peak corresponding to the stretching vibration of Fe–O bonds appeared at 578 cm^−1^ [40]. Such characteristics of the GO and MGO indicated that the proper magnetization was achieved on the GO surface and, thus, the MGO was ready for the in situ peptide synthesis matrix and the further FRET-based protease assay.

### 3.2. Evaluation of In Situ Peptide Synthesis on MGO

**Figure 2:**
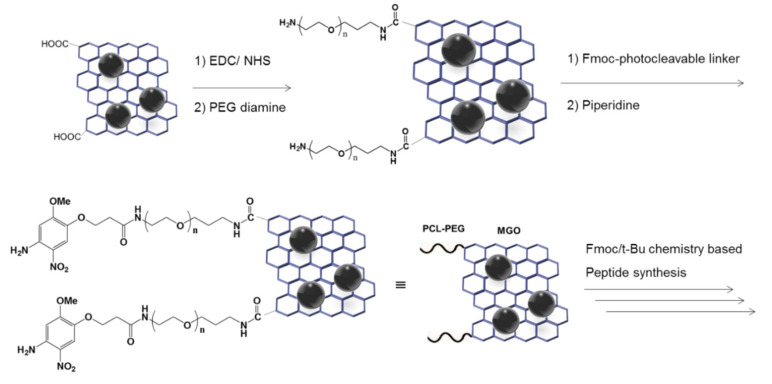
Coupling of polyethylene glycol (PEG) diamine and photocleavable linker (PCL) to MGO.

For in situ peptide synthesis on the MGO, the carboxyl group of the MGO was activated with EDC/NHS followed by coupling with poly(ethylene glycol) (PEG) diamine (Mn = 300, 500, 800, respectively); see (Figure 2). PEG diamine was incorporated to generate amino groups on the MGO and improve protease accessibility by acting as a spacer on the MGO surface. A Fmoc-PCL was subsequently coupled with the amino-functionalized MGO for the mass spectrometry analysis of released peptides. To evaluate the feasibility of Fmoc/*t*-Bu chemistry-based peptide synthesis on the MGO, two peptides specific to the thrombin and β-secretase (LVPRGS and EVNLDA, respectively) were synthesized on MGO matrices.

**Figure 3:**
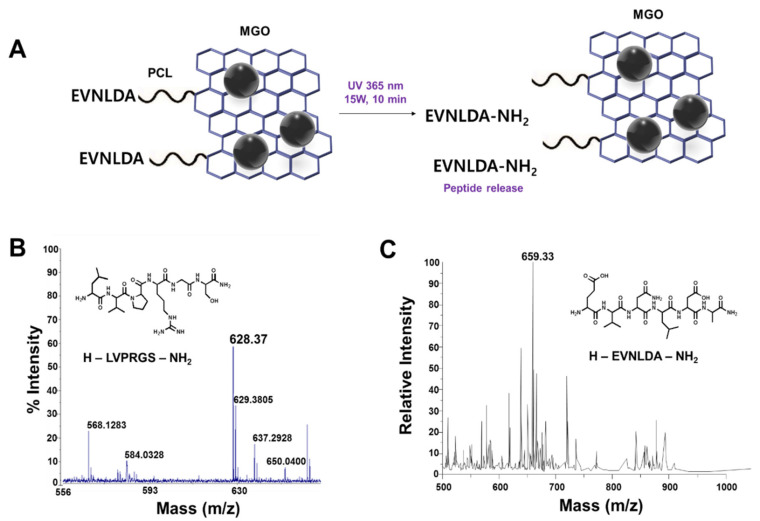
In situ peptide synthesis on MGO and mass spectrometry of the released peptides. (**A**) Schematic diagram of synthesized peptide on MGO and photocleavage reaction of β-secretase (EVNLDA) peptide on MGO. (**B**,**C**) MALDI-TOF analyses of H-thrombin(LVPRGS)-NH_2_ and H-EVNLDA-NH_2_ released from the MGO upon UV irradiation (365 nm).

After peptides were synthesized on the MGO matrices, the peptides were released by the cleavage of PCL upon UV irradiation (365 nm). Peptide sequences were confirmed by matrix assisted laser desorption/ionization time-of-flight mass spectrometry (MALDI-TOF MS) analysis after magnetic separation (Figure 3). Two strong peaks in the MALDI-TOF spectra at 628.37 and 659.33 shown in Figure 3B,C corresponded to the theoretical mass-to-charge (*m*/*z*, [M + H]^+^) values of H-LVPRGS-NH_2_ and H-EVNLDA-NH_2_ peptides, respectively. This result demonstrates that the MGO nano-composite materials with capability of direct peptide synthesis can be a potential platform for the FRET-based protease assay.

### 3.3. Optimization of PEG Spacer Length for Protease Activity Assay

**Figure 4:**
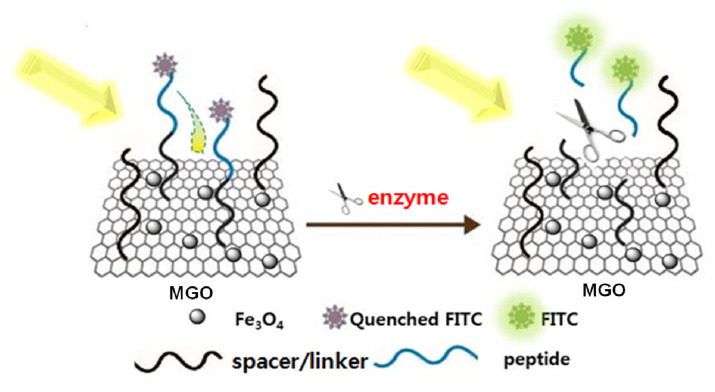
Illustration of MGO fluorescence resonance energy transfer (FRET) sensor based on fluorescence recovery induced by enzymatic cleavage.

A scheme of the working principle for the MGO FRET biosensor platform is illustrated in Figure 4. Protease-specific peptide was synthesized onto the MGO, followed by labeling of the *N*-terminal of the peptide with FITC as a fluorophore. Resonance energy transfer from the FITC to the MGO resulted in quenching of the fluorescence generated from the FITC. The protease cleaved a specific sequence of peptide-rendering FITC-labeled fragments released from the MGO surface, leading to fluorescence recovery of the FITC in solution. The degree of increase in fluorescence reflects the proteolytic activity.

**Figure 5:**
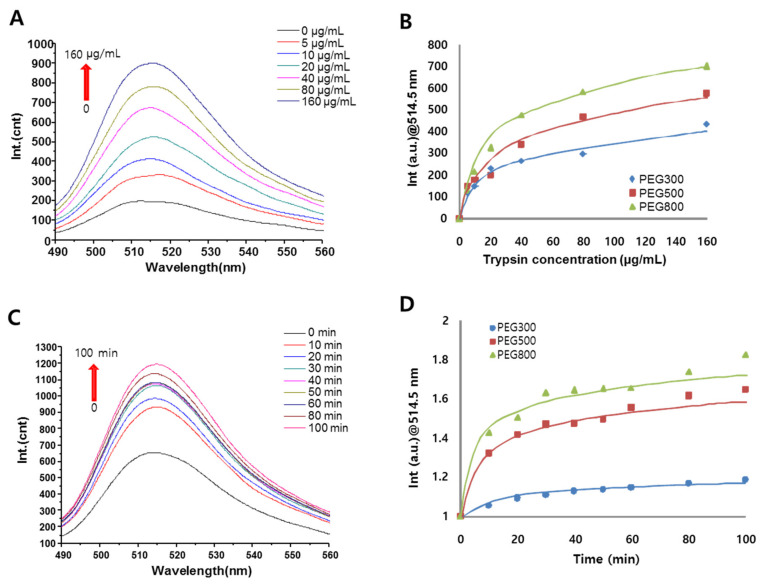
Fluorescence signals from fluorescein 5-isothiocyanate FITC-KKK PEGx-MGO biosensors upon tryptic peptide cleavage. (**A**) Fluorescence emission spectra of FITC-KKK-PEG_800_-MGO FRET biosensors in the presence of trypsin at concentrations of 0 to 160 μg/mL for 30 min. (**B**) Fluorescence intensity recovery (F/F_0_) at 514.5 nm depending on the PEG length in FITC-KKK-PEGx-MGO in response to different concentrations of trypsin. (**C**) Temporal fluorescence emission spectra of FITC-KKK-PEG_800_-MGO FRET biosensors from 0 to 100 min with 20 µg/mL of trypsin. (**D**) Time-dependent fluorescence intensity recovery (F/F_0_) at 514.5 nm depending on PEG length in FITC-KKK-PEGx-MGO with 20 µg/mL of trypsin.

The kinetic behaviors of FRET in the MGO-FITC platform incorporating FITC-KKK-PEGx in response to trypsin was first investigated as a function of concentration and reaction time. Figure 5A shows the fluorescence emission spectra of the FITC-KKK-PEG_800_-MGO in the presence of various concentrations of trypsin (0, 5, 10, 20, 40, 80, and 160 μg/mL). Trypsin-mediated cleavage of the peptide sequence KKK for 30 min resulted in the release of FITC-labeled fragments from the MGO. Fluorescence spectrophotometry data showed stronger fluorescence intensity at 514.5 nm as the concentration of trypsin increased. In Figure 5B, the normalized fluorescence intensity (F/F_0_) of the biosensors linked with PEG_300_-diamine, PEG_500_-diamine, and PEG_800_-diamine are plotted according to the concentrations of trypsin; fluorescence recovery improved with longer PEG chains. This result clearly indicated that increased space between the peptide and MGO matrix improved the recognition of trypsin by the tripeptide.

Figure 5C shows the fluorescence emission spectra of FITC-KKK-PEG_800_-MGO as a function of time (0 to 100 min) with 20 µg/mL of trypsin. Fluorescence intensity recovery was clearly observed within 10 min and gradually increased. To investigate the effect of the spacer length, temporal fluorescence intensity recovery (F/F_0_) was observed with 20 μg/mL of trypsin. Biosensors with the PEG_300_-diamine and PEG_500_-diamine spacers showed slower fluorescence recovery (Figure 5D). As evident in the fluorescence emission spectra, the ratio of increase in the intensity of FITC-KKK-PEG_800_-MGO (60% increase compared with normalized fluorescence count at 60 min) was 6-fold greater than that of FITC-KKK-PEG_300_-MGO (10% increase at 60 min, Figure 5D). On the basis of the above results, PEG_800_ was employed as a spacer between the MGO and peptide for improved sensitivity in further protease assays.

### 3.4. Evaluation of Thrombin Activity

**Figure 6:**
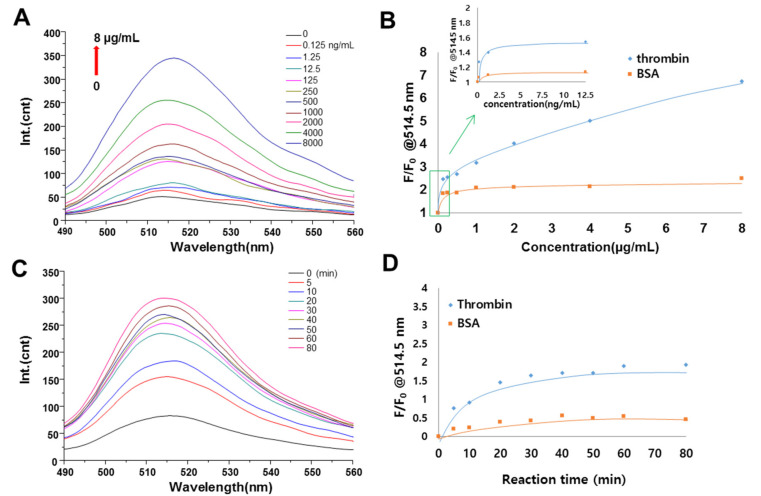
Fluorescence signals from FITC-LVPRGS-PEG_800_-MGO biosensors upon peptide cleavage by thrombin. (**A**) Fluorescence emission spectra of FITC-LVPRGS-PEG_800_-MGO FRET biosensors in the presence of thrombin at concentration of 0 to 8 μg/mL for 30 min. (**B**) Fluorescence intensity recovery (F/F_0_) at 514.5 nm from FITC-LVPRGS-PEG_800_-MGO in response to different concentrations of thrombin when compared with BSA. (**C**) Temporal fluorescence emission spectra of FITC-LVPRGS-PEG_800_-MGO FRET biosensors from 0 to 100 min at 2 μg/mL of thrombin. (**D**) Time-dependent fluorescence intensity recovery (F/F_0_) at 514.5 nm in FITC-LVPRGS-PEG_800_-MGO at 2 µg/mL of thrombin when compared to BSA (2 µg/mL).

The feasibility of the MGO FRET biosensor platform for thrombin detection using the FITC-LVPRGS-PEG_800_-MGO biosensor was explored. Fluorescence recovery of the biosensor in the presence of thrombin as a model biomarker took place by the thrombin cleavage of the peptide between Arg and Gly [41]. Various concentrations of thrombin from 0 to 8 µg/mL were incubated with the FITC-LVPRGS-PEG_800_-MGO biosensor for 30 min (Figure 6A). As a result, fluorescence intensified as the thrombin concentration increased. Normalized fluorescence intensity (F/F_0_) values of the FITC-LVPRGS-PEG_800_-MGO were plotted according to the concentrations of thrombin and bovine serum albumin (BSA) as a control protein (Figure 6B). The fluorescence intensity clearly increased as the concentration of thrombin increased, whereas BSA did not increase regardless of the concentration of BSA. Figure 6C shows the fluorescence emission spectra of FITC-LVPRGS-PEG_800_-MGO from 0 to 100 min with 2 µg/mL of thrombin. The temporal fluorescence intensity increased as the biosensor was challenged with protease solution. Furthermore, the fluorescence intensity recovery (F/F_0_) of the FITC-LVPRGS-PEG_800_-MGO was 4-fold greater in the presence of thrombin (2 μg/mL) when compared to BSA (2 μg/mL, Figure 6D). From these results, it could be concluded that thrombin was successfully assayed using the MGO FRET biosensor platform.

### 3.5. Evaluation of β-Secretase Activity

**Figure 7:**
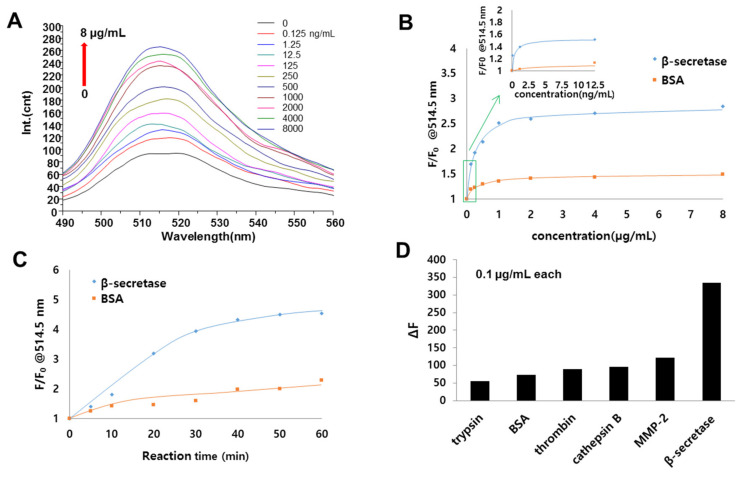
Fluorescence signals from FITC-EVNLDA-PEG_800_-MGO biosensors upon peptide cleavage by β-secretase. (**A**) Fluorescence emission spectra of FITC-EVNLDA-PEG_800_-MGO FRET biosensors in the presence of β-secretase at concentrations of 0 to 8 μg/mL for 30 min. (**B**) Fluorescence intensity recovery (F/F_0_) at 514.5 nm from FITC-EVNLDA-PEG_800_-MGO in response to different concentrations of β-secretase when compared to BSA. (**C**) Temporal fluorescence emission spectra of FITC-EVNLDA-PEG_800_-MGO FRET biosensors from 0 to 60 min at 2 μg/mL of β-secretase. (**D**) FRET activities of FITC-EVNLDA-PEG_800_-MGO for various proteins at a protein concentration of 0.1 μg/mL.

Finally, we investigated whether the MGO platform can be used for the detection of β-secretase, which is considered to be an important Alzheimer’s disease marker [42,43]. The MGO FRET biosensors were incorporated with the β-secretase-specific peptide (EVNLDA) [44]. Fluorescence recovery was achieved by the cleavage of the peptide between Leu and Asp by β-secretase, resulting in the release of FITC-EVNL. The fluorescence intensity increased with increasing concentration of β-secretase, as shown in Figure 7A. Figure 7B shows the normalized fluorescence intensities for FITC-EVNLDA-PEG_800_-MGO depending on the concentrations of target β-secretase and BSA as a control protein. This biosensor for β-secretase showed that the working concentration ranged from 0.125 ng/mL to 1.2 μg/mL. Similar to the thrombin assay, the fluorescence intensity increased as the concentration of β-secretase increased, whereas that of the BSA did not increase. Figure 7C shows the temporal fluorescence signals from the FITC-EVNLDA-PEG_800_-MGO for β-secretase. Fluorescence intensity gradually increased during the first 30 min and was then saturated after 40 min, whereas BSA did not increase over time. To investigate the substrate specificity of the peptide, FRET-EVNLDA-PEG_800_-MGO was challenged with various nonspecific proteins including proteases. The biosensor exhibited the capability of highly specific and selective detection of β-secretase over control enzymes and proteins including trypsin, BSA, thrombin, cathepsin B, and matrix metallopeptidase-2 (MMP-2) (Figure 7D). Overall, we summarized the protease assay results in the literature and compared with our results (Table 1). Even though the sensitivity is variable because of diverse proteolytic kinetics, the assay time (30 min) and the limit of detection (2 pM) from our results are comparable to the data from FRET-based pre-synthesized peptide/graphene complexed matrices in the literature. These results indicate that the FRET–MGO platform with in situ synthesized peptide EVNLDA holds a great promise for the development of a biosensor for the detection of Alzheimer’s disease. In addition, this sensing platform can easily be adapted to an automated synthesis system, thereby enabling the detection of various diseases.

## 4. Conclusions

In summary, we employed novel magnetic graphene oxide-based FRET biosensors (FITC-peptide-PEG-MGO biosensors) capable of in situ peptide synthesis and subsequent protease assays. Graphene oxide (GO) was prepared according to the improved Hummers’ method, and magnetic graphene oxide (MGO) was produced by the co-precipitation of Fe^2+^ and Fe^3+^ in an aqueous solution. The MGO was characterized by optical microscopy, a vibrating sample magnetometer (VSM), and UV/Vis and FT-IR spectroscopies. As confirmed by MALDI-TOF MS analysis, protease substrate peptides were successfully synthesized on the PCL-PEG-MGO. Fluorescence recovery from the FRET peptide MGO biosensors revealed that PEG_800_ as a linker had an optimal length for the tryptic activity assay. FRET peptide MGO biosensors were challenged with a solution of thrombin and β-secretase as biomarkers, and the proteases were assayed. In particular, β-secretase was successfully assayed using the FRET-EVNLDA-MGO biosensor in the concentration range of 0.125 ng/mL to 1.2 μg/mL. Overall, this MGO biosensor platform is ready to be automated and the peptide substrate will be diverse in a high throughput configuration. We envision that the FRET-MGO biosensor platform is applicable to the detection of other proteases through the use of various peptide substrates.

## Figures and Tables

**Table 1:** Comparison of protease assay performance on FRET-based graphene–peptide complexed matrices.

Specific Peptide	Target Protease	Assay Time	Limit of Detection	Reference
TTYADFIASGRTG-RRNAIHD (pre-synthesized)	Carboxypeptidase Y	8 h	1.0 × 10^−5^ U/μL	Wang et al. [30]
PLGVR (pre-synthesized)	MMP2 ^b^	2 h	0.19 ng/mL (3 pM)	Xi et al. [31]
CALNNSQNYPIVQK (pre-synthesized)	HIV-1 ^a^ protease	30 min	1.18 ng/mL (109 pM)	Zhang et al. [45]
GKGGLVPRGSGC, GPLGVRGC, GKGGLVPRGSGK (pre-synthesized)	MMP2, thrombin	60 min	MMP2: 1.0 nM; Thrombin: 0.5 nM	Li et al. [46]
EVNLDA (in situ synthesized)	β-secretase	30 min	0.125 ng/mL (2 pM)	Kim and Lee et al. (This study)

^a^ human immunodeficiency virus-1; ^b^ matrix metalloproteinase 2.
